# Association between anemia and cognitive decline among Chinese middle-aged and elderly: evidence from the China health and retirement longitudinal study

**DOI:** 10.1186/s12877-019-1308-7

**Published:** 2019-11-12

**Authors:** Tingting Qin, Mingming Yan, Zhen Fu, Yating Song, Wanrong Lu, A’dan Fu, Ping Yin

**Affiliations:** 1 Department of Biliary-Pancreatic Surgery, Affiliated Tongji Hospital, Tongji Medical College, Huazhong University of Science and Technology, Wuhan, 430030 Hubei China; 2 Department of Epidemiology and Biostatistics and State Key Laboratory of Environment Health, School of Public Health, Tongji Medical College, Huazhong University of Science and Technology, 13 Hangkong Rd, Wuhan, 430030 Hubei China; 3 Department of Nursing, The Central Hospital of Wuhan, Tongji Medical College, Huazhong University of Science and Technology, 26 Shengli Rd, Wuhan, 430014 Hubei China

**Keywords:** Cognitive decline, Anemia, Hemoglobin, Relationship

## Abstract

**Background:**

Our objective was to characterize the relationship of anemia and hemoglobin concentrations with cross-sectional cognitive functions and changes in cognitive functions over 2 years in a large sample of Chinese middle aged and elderly.

**Methods:**

Ten thousand nine hundred eighteen adults aged 45 years or older participating in the China Health and Retirement Longitudinal Study (CHARLS) were used for cross-sectional analyses and 9324 were used for longitudinal analysis. Cognitive functions were assessed by memory recall (episodic memory), mental status (TICS), and global cognitive function at baseline survey (Visit 1) and first follow-up survey (Visit 2). The lower the cognitive test score, the worse the cognitive function. Anemia was defined as hemoglobin concentrations lower than 13 g/dl for men and lower than 12 g/dl for women. Adjusted multivariate regression analyses were used to explore the relationships of different cognitive domains with anemia and hemoglobin concentration.

**Results:**

Overall, the prevalence of anemia was 12.86% and the mean hemoglobin concentration was 14.37 ± 2.20 g/dl. After adjusting for socio-demographic and health-related covariates, the cross-sectional association between anemia and global cognitive function [*β* (95%*CI*) = − 0.49(− 0.69~ − 0.29)], episodic memory [*β* (95%*CI*) = − 0.14(− 0.23~ − 0.05)], and TICS [*β* (95%*CI*) = − 0.23(− 0.38~ − 0.08)] were significant and did not differ by gender. The hemoglobin concentration was also associated with global cognitive function among the whole sample (*P* < 0.05 for all). The longitudinal analyses showed global cognitive function and episodic memory were associated with anemia independent of covariates (*P* < 0.05 for all). Sensitivity analyses further provided significant results showing the association between anemia and cognition decline (*P* < 0.05).

**Conclusion:**

There was a cross-sectional and longitudinal association between anemia and accelerated decline in cognitive functions in Chinese middle-aged and elderly. This suggests that anemia and low hemoglobin concentrations are independent risk factors of cognitive decline.

## Background

With the intensification of aging all over the world and increased life expectancies, cognitive decline as the main precursor of dementia has become a serious human, social, and economic burden among the middle-aged and elderly than before [1] .Although cognitive decline can be observed in the normal aging process, the different trajectories of age-related cognitive decline between individuals suggests there is subject-specific risk of cognitive impairment and dementia development along with pathological aging [2]. Concerns of cognitive decline commonly manifest in healthy older adults [3], and have been estimated to appear in approximately from 25 to 50% of the community-dwelling older adults [4]. Progressive decline of cognitive function is an early risk factor for dementia [5], and is the primary clinical manifestation of Alzheimer’s disease (AD) [6]. Thus, increasing researches have examined the possible modifiable risk factors of rapid cognitive decline and to prevent the incidence of dementia or AD.

Among medical conditions accompanying with the cognitive decline in the process of pathological aging, anemia is an important concern. Studies examining the effect of anemia on cognitive decline were mainly motivated by some specific samples such as the hospitalized elderly [7], those with end-stage renal disease [8], heart failure [9], malignancy [10] and the fingdings indicated that anemia was highly associated with cognitive impairement. In addition, among the general community-dewelling older adult population, amemia was also found to be a indenpendant risk factor of cognitive decline. For example, results from two meta-analyses reported anemia (defined as hemoglobin concentrations lower than 13 g/dl for men and lower than 12 g/dl for women [11]) was associated with the global cognitive decline as well as the incidence of dementia and the reduction of executive functions [12, 13]. Moreover, epidemiological evidence indicates that low hemoglobin concentrations were associated with increased risk of incident Alzheimer disease (AD). For instance, the incidence of cognitive decline and Alzheimer’s disease is considered to be increased about twofold amongst patients with anemia [14]. Evidence also existed that the incident dementia 3 years later was associated to anemia among older persons aged 75–95 years [15]. Consistent with anemia, low hemoglobin concentration was also reported to be associated with cognitive impairment and some specific cognitive domians inversely [16–18]. However, there were still some literatures which found that anemia was not significantly associated with cognitive decline after adjusting for potential confounding factors. Tamura M et al found the association between anemia and cognitive decline was no longer significant after adjusting for multi-covariates, such as age, race, gender, education, region, kidney function, diabetes, hypertension, hyperlipidemia, coronary heart disease, stroke, depressive symptoms, tobacco and alcohol use, health status, and C-reactive protein level in a 19,701 community-dwelling adults sample [19]. Lucca et al found no-significant relationship between anemia and cognitive decline after adjusting for some health problems such as hypertension, diabetes, heart failure, myocardial infarction, respiratory failure, and neurologic disorders [20]. The diverse study samples (e.g., age, income, education, and geographic location), specific study designs, different analytical methods, and adjustment for confounders may partly account for these controversial findings. So, it is still necessary to further explore the relationship between anemia and cognitive decline because identification of individuals at risk for rapid cognitive decline is a fundamental prerequisite for the early intervention of dementia or AD [21]. Besides, the lack of studies regarding risk of anemia and its association with cognitive functioning on large community-dwelling elderly samples, particularly in China, was also motivated this research.

In order to provide more evidence for explaining the relationship between anemia and cognitive decline, we sought to identify this relationship with better consideration of wider lifespan containing middle aged adults and possible confounders using a national representative sample - China Health and Retirement Longitudinal Study (CHARLS). We hope a large sample both including the middle-aged and the elder adults may complete the gaps in the prior literature to improve the understanding of the anemia-cognition association. We hypothesize that anemia is associated with cognitive impairment cross-sectionally. We also hypothesize that the anemia is associated with cognitive decline after 2 years follow-up independent of potential confounders. Besides, we suppose that lower hemoglobin concentration is associated with poor cognitive performance in a linear trend. Cognitive funtion has abundant domains, especially the performance on episodic memory task attracted most interests. And we will include and evaluate these cognitive domians and make full evalutions of the relationship between anemia and cognition performance. Considering different definitions of anemia by gender and the fact that women are more vulnerable to anemia than men, we additionally sought to estimate the gender-specific effects on this relationship. We believe this issue is important because identification of reversible causes and mechanisms of cognitive decline could help developing useful intervention approaches to prevent the adults’ cognitive decline.

## Methods and measurements

### Study sample

The current study was originated from the China Health and Retirement Longitudinal Study (CHARLS), a nationally representative cohort study of longitudinal survey with participants aged 45 years or older and run by the National School for Development (China Center for Economic Research) at Peking University (PKU). The sampling method of the CHARLS has been described in detail elsewhere [22]. The baseline survey (Visit 1) was conducted in 2011–2012 and the second follow-up survey (Visit 2) was conducted in 2013. Information was gathered using face-to-face computer-assisted personal interview (CAPI). The cohort contained 17,707 respondents with the respondent rate above 80%.

Cognitive assessment was first performed at baseline survey (Visit 1). After excluding those have missing values of cognitive assessment (*N* = 1667) and those without hemoglobin assessment (*N* = 6117), the eligible participants enrolled in the analytic cohort for cross-sectional analysis were 10,918. Then, the cognitive assessment was re-performed at second follow-up survey (Visit 2) and severed as the outcome for longitudinal analyses. Among the 10,918 participants of baseline, 973 dropped out from Visit 2 and 621 missed the cognitive assessments, leaving 9324 participants in the analytic cohort for longitudinal analyses. The informed consents were obtained from all participants and this study was approved by the biomedical ethics committee of Beijing University.

### Cognitive assessment

Similarly to the cognitive assessments used in the American Health and Retirement Study, three cognitive domains measuring the dimensions of orientation and attention, word recall, and visuospatial abilities were used and measured both at Visit 1 and Visit 2 by trained examiners [23]. The first was memory recall based on a respondent’s ability to immediately repeat ten Chinese nouns in random order just read to him/her (immediate recall) and to recall the same list of nouns 5 minutes later (delayed recall) [24]. The average of immediate and delayed recall scores formed a single **episodic memory** score that ranged from 0 to 10. **Episodic memory** represents one’s memory for autobiographical events [25]. The second was mental status based on some components of the mental status questions of the Telephone Interview of Cognitive Status (TICS) battery- a well-established measure to capture one’s mental status or intactness [26]. The measurements were carried out by asking the participants to answer the following questions - serial 7 subtractions from 100 (up to five times), naming today’s date (day, week, month, year, and season), self-rated memory, and to redraw a picture shown to him/her. The right answers were summed up to a single **TICS** score that ranges from 0 to 11. The **TICS** score has been previously used to describe one’s orientation/attention abilities and visuospatial ability [27, 28]. The last was an overall measurement of respondent’s cognitive function that aggregated the above two measurements of episodic memory and TICS score into a single **global cognitive function** score ranging from 0 to 21. The global cognitive function was considered to be the primary interest outcome. This cognitive assessment has been used in previous publications [27–31].

### Blood measurement and Anemia definition

The blood sample was collected on all eligible participants every two investigations, so the blood-related variables analyszed in the present analysis was obtained from the baseline survey during 2011–2012. Blood sample were collected after an overnight fast by medically-trained staff at centralized locations [the district Centers for Disease Control (CDC) for urban areas, and the county CDC stations or the town/village health centers for rural areas]. Venous blood was obtained for the complete blood counts (CBC) test, including hemoglobin concentrations (g/dl) and mean corpuscular volume (MCV, fl), and measured on automated analyzers available at centralized locations. Other fresh venous blood samples were immediately separated into plasma and buffy coat and stored frozen at − 20 °C before transported to the Chinese Centers for Disease Control in Beijing within 2 weeks. The transported blood sample was placed in a deep freezer and stored at − 80 °C for the detection of other blood biochemical, such as serum creatinine, glucose, blood lipid, and C-reactive protein (CRP). The detection procedures were performed at the Youanmen Center for Clinical Laboratory of Capital Medical University. The serum creatinine was measured by rate-blanked and compensated Jaffe creatinine method. The blood lipids including total cholesterol and high density lipoprotein cholesterol were measured by enzymatic colormetric test. And the C-reactive protein was measured by immunoturbidimetric assay. According to the World Health Organization criteria, anemia was defined as hemoglobin concentrations lower than 13 g/dl for men and lower than 12 g/dl for women [11].

### Other covariates

Potential covariates including demographic variables and health-related factors were assessed at the baseline survey, mainly including age, gender, education, cigarette smoking, alcohol drinking, and comorbidities. Education level was categorized as five mutually exclusive groups: illiterate, elementary school, middle school, high school, and graduate or above). Marital status was categorized into married, divorced, widowed and never married. Smoking status and alcohol consumption were categorized as “former”, “current”, and “never”. Body mass index (BMI) was derived from direct height and weight basing on the standard formula: kg/m^2^. Hypertension was determined as self-reported physician diagnosis, medication use, or systolic blood pressure ≥ 140 mmHg, or diastolic blood pressure ≥ 90 mmHg. Diabetes was defined as self-reported physician diagnosis, medication use, fasting glucose≥126 mg/dl, or non-fasting glucose ≥200 mg/dl. Abdominal adiposity was defined as abdomen circumference equal to or greater than 90 cm for men and equal to or greater than 80 cm for women. The dyslipidemia was defined as triglyceride equal to or greater than 200 mg/dl, or cholesterol equal to or greater than 240 mg/dl, or high-density lipoprotein equal to or greater than 40 mg/dl, or low density lipoprotein equal to or greater than 160 mg/dl. Chronic pain was assessed by asking the participants if they often suffered from pain, and only those with moderate or severe pain were classified as suffering from chronic pain. The glomerular filtration rate (GFR, reflects renal function) was estimated using the Cockcroft-Gault equation [32]. Depressive symptoms were assessed by the Center for Epidemiologic Studies Short Depression Scale (CES-D 10) [33]. Considering cognitive condition can be influenced by some clinical status, we also collected the history of comorbidities, including heart problems, stroke, cancer, liver disease, chronic lung disease, renal disease and other diseases, based on self-reporting and medicine use [10, 34, 35].

### Statistical analysis

Baseline characteristics of the study sample were presented across anemia and non-anemia groups. The comparisons between two groups were conducted by χ^2^-test or Wilcoxon rank sums tests for categorical variables, and Student’s *t*-test for continuous variables. Age-adjusted comparison of each cognitive domain was done using the analyses of covariance (ANCOVA). Multivariate regression analyses were conducted to assess the relationship of anemia or low hemoglobin concentrations with cognitive function measured at Visit 1 and the relationship of anemia with cognitive decline measured at Visit 2. The cognitive decline was estimated as the difference score of cognitive performance measured at Visit 2 and Visit 1. Regression coefficient (*β*) and 95% confidence interval (*CI*) were then computed with the global cognitive function, episodic memory, and TICS score being the outcome in regression models, separately.

Considering cognitive function was a highly age-dependent assessment, the initial statistical model (Model 1) was used to estimate the raw association between anemia and cognitive function only adjusted for age. The second statistical model (Model 2) was adjusted for demographic and health-related factors, including education, marital status, BMI, cigarette smoking, alcohol consumption, C-reactive protein, total cholesterol, high density lipoprotein cholesterol on the basis of Model 1. The third statistical model (Model 3) consisted of stepped entry of disease-related factors, including chronic pain and related comorbidities on the basis of Model 2. Given that hemoglobin concentration can be influenced by renal function and high mean corpuscular volume (MCV) level [36, 37], we additionally adjusted estimated GFR and MCV in model 3 in an attempt to adjust for their confounding effects.

In view of the complicated association between anemia and cognitive performance, three sensitivity analyses were conducted to further confirm the relationship on the basis of Model 3. Firstly, considering the unbalanced distribution of baseline covariates between anemia and non-anemia participants, we applied the propensity score methodology- defined as the conditional probability of being treated when given the covariates, to control the influence of covariates [38]. Therefore, the bias of estimates can be reduced. We matched anemic participants at baseline survey 1:4 to non-anemic participants using Mahalanobis metric matching within a caliper width of 0.2 of the standard deviation of the logit of the propensity score [39]. After matching, we applied propensity score as the covariate and estimated the association between anemia and cognitive function on the basis of model 3. Secondly, participants who had low (< 80) or high (> 100) MCV were excluded to eliminate the impact of microcytic or macrocytic anemia. Thirdly, participants who had low BMI (< 18.5 kg/m^2^), indicating underweight individuals according to the WHO, were excluded to eliminate the impact of malnutrition [40].

Due to the different definitions of anemia by gender, we decided to present all results stratified by gender and explore whether there was gender-specific difference in regards to the relationship of anemia and cognitive decline. In order to take consideration of the complex survey design and the differential response rates of CHARLS, all statistical processes were performed using relevant PROC SURVEY procedures in SAS (version 9.4, SAS Institute Inc., Cary, NC, USA) Reported *p* values were two sided, and a predetermined level of *P* < 0.05 was considered statistically significant.

## Results

### General description

**Fig. 1:**
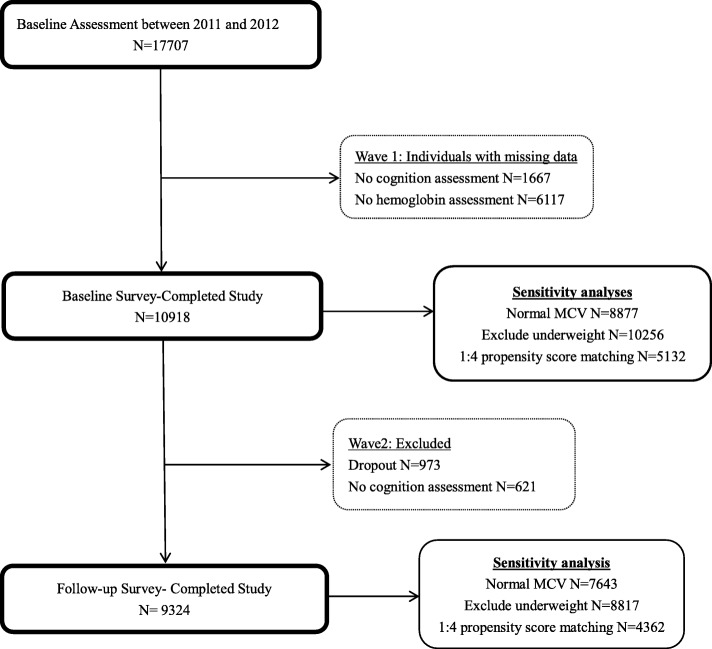
Flow chart of patients through the study

A total of 10,918 participants (4999 men and 5919 women) with a mean age of 59.2 (*SD* = 9.66) years ((58.81 ± 9.36 years in non-anemia group and 61.31 ± 11.26 in anemia group) were eligible for the final cross-sectional study. Study sample selection is described in Fig. 1. The mean hemoglobin concentration was 14.37(*SD* = 2.20) g/dl (range = 5.40~27.87 g/dl). 1404 (12.86%) were characterized as anemic (11.1% for men and 14.25% for women). The mean score of global cognitive function was 10.37 (*SD* = 4.32), the TICS was 7.22 (*SD* = 3.03), and the episodic memory was 3.79(*SD* = 1.51). Compared to the participants without anemia, the anemic ones were more likely to be women and older. In addition, those with anemia were also more likely to have lower education level, lower BMI, hypertension, to be alcohol users, and suffer from depressed than the non-anemic ones. Table 1 displayed the characteristics of study sample according to anemia status.

**Table 1:** Baseline participant characteristics by anemia status^a^, CHARLS (Visit 1, 2011-2012y, *N* = 10,918)

Variable	Total (N = 10,918)	Non-anemia (*N* = 9513)	Anemia (*N* = 1404)	*p-*value^b^
Age (years), mean (SD)	59.13 (9.66)	58.81 (9.36)	61.31 (11.26)	< 0.0001
Male, %(n)	45.79 (4999)	46.72 (4444)	39.53 (555)	< 0.0001
Hemoglobin (g/dL), mean (SD)	14.37 (2.20)	14.82 (1.93)	11.27 (1.24)	< 0.0001
Alcohol consumption, %(n)				0.0026
Never	30.67 (3349)	31.13 (2961)	27.64 (388)	
Occasional	7.72 (843)	7.83 (745)	6.98 (98)	
Current	61.60 (6726)	61.04 (5807)	65.38 (918)	
Smoking status, %(n)				< 0.0001
Never	61.01 (6661)	60.20 (5727)	66.45 (933)	
Occasional	9.13 (997)	9.19 (874)	8.76 (123)	
Current	29.86 (3260)	30.61 (2912)	24.79 (348)	
Marital status, %(n)				0.0643
Married	88.64 (9678)	88.84 (8451)	87.32 (1226)	
Divorced	0.64 (70)	0.69 (66)	0.28 (4)	
Widowed	9.99 (1091)	9.74 (927)	11.68 (164)	
Single	0.72 (79)	0.73 (69)	0.71 (10)	
Education, %(n)				< 0.0001
Illiterate	28.60 (3132)	27.83 (2647)	34.54 (485)	
Elementary school	40.91 (4467)	40.65 (3867)	42.66 (599)	
Middle school	19.88 (2171)	20.47 (1947)	15.95 (224)	
High school	10.01 (1093)	10.56 (1005)	6.27 (88)	
Graduate or above	0.50 (55)	0.49 (47)	0.57 (8)	
BMI degree, %(n)				< 0.0001
Underweight	5.93 (552)	5.28 (426)	10.29 (126)	
Normal weight	53.04 (4933)	52.36 (4228)	57.55 (705)	
Overweight	30.05 (2795)	30.89 (2494)	24.49 (300)	
Obesity	10.98 (1021)	11.48 (927)	7.67 (94)	
CES-D 10, mean (SD)	8.61 (6.42)	8.53 (6.39)	9.20 (6.56)	0.0003
Hypertension, %(n)	4.53 (495)	4.76 (453)	2.99 (42)	0.0029
Diabetes, %(n)	15.18 (1657)	15.18 (1444)	15.17 (213)	0.9936
Abdominal Adiposity, %(n)	45.46 (4963)	46.16 (4391)	40.74 (572)	< 0.0001
Suffering chronic pain, %(n)	25.95 (2829)	25.69 (2441)	27.62 (387)	0.1242
Dyslipidemia,%(n)	44.09 (4814)	45.29 (4308)	36.04 (506)	< 0.0001
Hs-CRP (mg/l),median (IQR)	1.06 (0.55~2.27)	1.07 (0.58~2.26)	0.94 (0.51~2.43)	0.6475
Mean corpuscular volume, mean (SD)	90.56 (8.58)	91.22 (7.76)	86.11 (11.93)	< 0.0001
Hdl Cholesterol (mg/dl), mean (SD)	50.93 (15.16)	50.72 (15.07)	52.33 (15.68)	< 0.0001
eGFR, mean (SD)	92.54 (23.51)	92.61 (22.88)	92.11 (27.38)	0.3577

*Hs-CRP* High-sensitivity of C-reactive protein, *eGFR* Estimated glomerular function rate, *CES-D 10* 10-item Center for Epidemiologic Studies Depression Scale score

Notes: ^a^ Anemia was defined as hemoglobin concentrations lower than 13 g/dl for men and lower than 12 g/dl for women;

^b^compared between anemia and non-anemia group

**Fig. 2:**
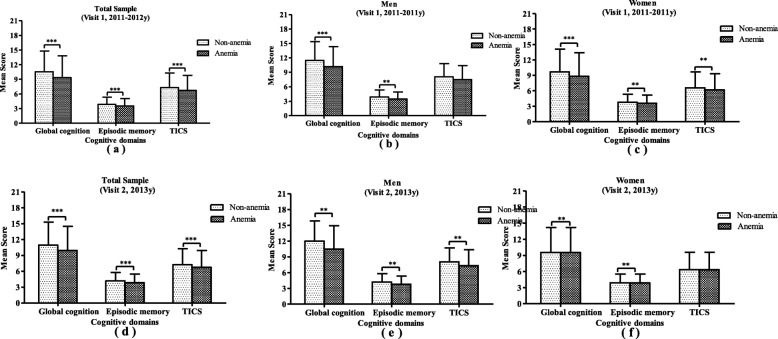
Age-adjusted comparison of Cognitive measures between anemic and non-anemic groups by gender. The mean value of Cognitive Test Scores measured at baseline survey (2011-2012y) between anemia and non-anemia group is illustrated separately for global cognition, episodic memory, and TICS among the total sample (Panel **a**), among men (Panel **b**), and among women (Panel **c**). The mean value of Cognitive Test Scores re-measured at follow-up survey (2013y) between anemia and non-anemia group is illustrated separately for global cognition, episodic memory, and TICS among the total sample (Panel **d**), among men (Panel **e**), and among women (Panel **f**). *** means *p* < 0.0001; ** means *p* < 0.001

For the entire sample, the mean score of global cognition measured at baseline survey was 9.36 (*SD* = 4.46) in the anemia group, which was significantly lower than the non-anemia group [mean (*SD*) =10.52(4.28), *P <* 0.001]. Those with anemia also had lower score on episodic memory [mean (*SD*): 3.54(1.51) vs 3.85(1.51), *p* < 0.001] and TICS [mean (*SD*): 6.71(3.10) vs 7.29(3.01), *P* < 0.001]. The anemic ones had lower performance on the cross-sectional cognitive function as well as longitudinal cognitive function without gender specific difference. These details are shown in Fig. 2.

### Cross-sectional relationship between anemia and cognition

Adjusted cross-sectional associations between cognition and anemia are displayed in Table 2. After adjusting for age (Model 1), anemia was cross-sectionally associated with lower cognitive scores on three cognitive measures among the entire sample. After adjusting for additional socio-demographic (Model 2) and health-related variables (Model 3), the cognitive measures were also significantly associated with anemia. However, when further estimated the association for men and women separately, only global cognitive function and episodic memory were consistently associated with anemia without being affected by the study sample. Women with anemia showed significantly worse performance on TICS, while men did not. These results are presented in Table 2.

**Table 2:** Gender-stratified relationship of baseline cognitive measures with anemia^a^ and hemoglobin, CHARLS (Visit 1, 2011-2012y, *N* = 10,918)

Outcome variable	Analytic Model	All^d^		Male		Female	
		*β* (95%CI)	*p-*value	*β* (95%CI)	*p-*value	*β* (95%CI)	*p-*value
Anemia^b^							
Global Cognition	Model1	− 0.65(− 0.87, − 0.42)	< 0.001	− 0.63(− 0.96, − 0.30)	0.003	− 0.73(− 1.03, − 0.42)	< 0.001
	Model2	− 0.45(− 0.64, − 0.25)	< 0.001	− 0.33(− 0.64, − 0.03)	0.030	− 0.55(− 0.81, − 0.29)	< 0.001
	Model3	−0.49(− 0.69, − 0.29)	< 0.001	− 0.40(− 0.71, − 0.10)	0.010	− 0.56(− 0.82, − 0.29)	< 0.001
Episodic Memory	Model1	− 0.20(− 0.29, − 0.11)	< 0.001	− 0.21(− 0.35, − 0.08)	0.002	− 0.19(− 0.31, − 0.07)	0.002
	Model2	− 0.14(− 0.23, − 0.06)	0.001	− 0.14(− 0.27, − 0.01)	0.033	− 0.15(− 0.26, − 0.03)	0.012
	Model3	− 0.14(− 0.23, − 0.05)	0.002	−0.14(− 0.28, − 0.01)	0.037	−0.14(− 0.25, − 0.02)	0.023
TICS	Model1	−0.28(− 0.44, − 0.11)	0.001	−0.22(− 0.47, 0.02)	0.076	−0.37(− 0.59, − 0.15)	0.001
	Model2	−0.16(− 0.30, − 0.02)	0.028	−0.03(− 0.25, 0.19)	0.787	−0.26(− 0.45, − 0.07)	0.007
	Model3	−0.23(− 0.38, − 0.08)	0.002	−0.11(− 0.33, 0.12)	0.360	−0.33(− 0.52, − 0.13)	0.001
Hemoglobin Concentration (g/dL)^c^							
Global Cognition	Model1	0.038(0.002, 0.074)	0.039	0.08(0.03, 0.13)	0.001	0.02(−0.04, 0.07)	0.545
	Model2	0.037(0.005, 0.069)	0.022	0.04(−0.01, 0.09)	0.080	0.04(−0.01, 0.09)	0.060
	Model3	0.042(0.011, 0.074)	0.011	0.05(0.01, 0.10)	0.036	0.04(−0.01, 0.09)	0.068
Episodic Memory	Model1	0.012(−0.002, 0.026)	0.083	0.02(−0.01, 0.04)	0.059	0.01(− 0.01, 0.03)	0.482
	Model2	0.012(−0.002, 0.026)	0.082	0.01(−0.01, 0.03)	0.262	0.01(− 0.01, 0.03)	0.146
	Model3	0.012(−0.002, 0.026)	0.087	0.01(−0.01, 0.03)	0.269	0.01(− 0.01, 0.03)	0.182
TICS	Model1	−0.004(− 0.030, 0.023)	0.788	0.03(− 0.01, 0.07)	0.077	−0.02(− 0.06, 0.01)	0.211
	Model2	0.001(− 0.022, 0.025)	0.908	0.01(− 0.03, 0.04)	0.622	−0.01(− 0.03, 0.03)	0.924
	Model3	0.008(− 0.016, 0.031)	0.517	0.02(− 0.02, 0.05)	0.325	0.01(− 0.03, 0.04)	0.862

*BMI* Body mass index, *MCV* Mean corpuscular volume, *eGFR* Estimated glomerular function rate, *CI* Confidence interval, *TICS* Telephone Interview of Cognitive Status.

*Notes:*^a^ Anemia was defined as hemoglobin concentrations lower than 13g/dl for men and lower than 12g/dl for women;

^b^The dichotomous anemia being the independent variable;

^c^The continuous hemoglobin concentration (g/dL) being the independent variable;

^d^When analyzed for the whole sample, gender was included as covariate for model 1 to model 3;

**Fig. 3:**
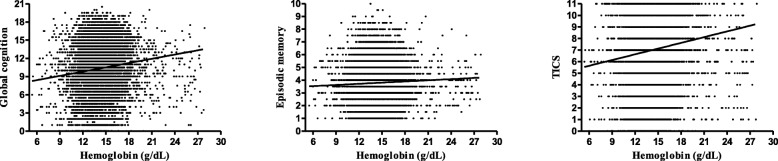
The relationship between Cognitive measures and hemoglobin concentration (g/dL). Panel a presents the relationship of Global Cognition with hemoglobin concentration among the whole sample (*r* = 0.120, *P* < 0.001). Panel b presents the relationship of Episodic Memory with hemoglobin concentration among the whole sample (*r* = 0.045, *P* < 0.001); Panel c presents the relationship of TICS with hemoglobin concentration among the whole sample (*r* = 0.114, *P* < 0.001)

Model 1 adjusted for age;

Model 2 adjusted for age, education level, marital status, BMI, cigarette smoking status, alcohol consumption, C reactive protein, total cholesterol, high density lipoprotein cholesterol;

Model 3 adjusted for age, education level, marital status, BMI, cigarette smoking status, alcohol consumption, C reactive protein, total cholesterol, high density lipoprotein cholesterol, chronic pain, MCV, eGFR and associated comorbidities (cancer, kidney, stroke, heart, lung, hypertension, diabetes );

When it comes to hemoglobin concentration, Fig. 3 shows a slight but significant linear relationship between global cognitive function and hemoglobin based on the entire sample (*r* = 0.120, *P* < 0.001). The regression analyses also showed that higher hemoglobin concentration were associated with better cognitive performance [*β* (95%*CI*) =0.038(0.002, 0.074), *P* = 0.039 for model 1; *β* (95%*CI*) = 0.037(0.005, 0.069), *P* = 0.022 for model 2; *β* (95%*CI*) = 0.042(0.011, 0.074), *P* = 0.011 for model 3]. However, when it refers to specific cognitive measures, the episodic memory and TICS score showed no significant relationship with hemoglobin concentration (all *P* > 0.05).

### Longitudinal relationship between anemia and cognition

There were 923 participants who did not return for Visit 2 and 621 did not complete any follow-up cognitive assessment, leaving 9324 participants for the longitudinal analyses. After making adjustments for baseline cognitive function and age, the decline of global cognitive function, episodic memory and TICS score were significantly associated with anemia. The significant associations between anemia and TICS score can be found after adjusting for demographic characters (Model 1) and health-related factors (Model 2). But after adjusting for the disease-related factors (model 3), the association between anemia and TICS score was no longer significant. In contrast, the association between anemia and TICS score was not significant in any analytical model among the women. However, the global cognitive function and episodic memory were associated with anemia consistently with stepwise adjusting for covariates among all analytical sample. These results are presented in Table 3.

**Table 3:** Gender-stratified longitudinal relationship between cognitive measures and anemia^a^, CHARLS (Visit 2, 2013y, *N* = 9324)

Outcome variable	Analytic Model	All^b^		Male		Female	
		*β* (95%CI)	*p-*value	*β* (95%CI)	*p-*value	*β* (95%CI)	*p-*value
Global Cognition	Model1	−0.62(−0.87, −0.38)	< 0.001	−0.92(− 1.29, − 0.55)	< 0.001	−0.50(− 0.84, − 0.17)	0.003
	Model2	− 0.39(− 0.61, − 0.18)	< 0.001	−0.59(− 0.92, − 0.25)	0.001	−0.30(− 0.58, − 0.02)	0.039
	Model3	− 0.24(− 0.43, − 0.04)	0.019	−0.50(− 0.81, − 0.19)	0.002	−0.09(− 0.35, 0.17)	0.496
Episodic Memory	Model1	−0.25(− 0.35, − 0.15)	< 0.001	−0.27(− 0.43, − 0.11)	0.001	−0.23(− 0.36, − 0.10)	0.001
	Model2	− 0.19(− 0.28, − 0.09)	< 0.001	−0.19(− 0.34, − 0.04)	0.013	−0.18(− 0.31, − 0.06)	0.003
	Model3	− 0.16(− 0.26, − 0.07)	0.001	−0.18(− 0.33, − 0.03)	0.017	−0.15(− 0.27, − 0.03)	0.016
TICS	Model1	− 0.26(− 0.44, − 0.08)	0.004	−0.45(− 0.71, − 0.18)	0.001	−0.21(− 0.45, 0.04)	0.099
	Model2	−0.13(− 0.29, 0.03)	0.117	− 0.26(− 0.51, − 0.01)	0.039	−0.07(− 0.27, 0.14)	0.542
	Model3	−0.04(− 0.19, 0.11)	0.634	− 0.23(− 0.47, 0.01)	0.053	0.08(− 0.12, 0.27)	0.444

*BMI* Body mass index, *MCV* Mean corpuscular volume, *eGFR* Estimated glomerular function rate, *CI* Confidence interval, *TICS* Telephone Interview of Cognitive Status

*Notes:*^a^ Anemia was defined as hemoglobin concentrations lower than 13 g/dl for men and lower than 12 g/dl for women; All analytical models were conducted with the Cognitive Test Scores of Visit 2 as the outcome and the dichotomous anemia as the independent variable;

^b^When analyzed for the whole sample, gender and was included as covariate for model 1 to model 3;

Model 1 adjusted for age and baseline hemoglobin concentration;

Model 2 adjusted for age, education level, marital status, BMI, cigarette smoking status, alcohol consumption, C reactive protein, total cholesterol, high density lipoprotein cholesterol, and baseline hemoglobin concentration;

Model 3 adjusted for age, education level, marital status, BMI, cigarette smoking status, alcohol consumption, C reactive protein, total cholesterol, high density lipoprotein cholesterol, chronic pain, associated comorbidities (cancer, kidney, stroke, heart, lung, hypertension, diabetes), MCV, eGFR, and baseline hemoglobin concentration;

### Sensitivity analysis

After propensity score matching, there were 1404 anemia and 3804 non-anemia participants left in the first situation of sensitivity analysis. The sample characteristics between anemia and non-anemia groups were post-balanced. The cross-sectional association between three cognitive measures and anemia remained significant after propensity score matching, excluding subjects who had abnormal mean corpuscular volume, as well as excluding the subjects who had low BMI. However, in the longitudinal analysis, except for the TICS score, the other two measures- global cognitive function and episodic memory were consistently significantly associated with anemia in all three sensitivity analyses. Details are shown in Table 4.

**Table 4:** Sensitivity analyses of the relationship between Cognitive measures and Anemia^a^

Methods	Outcome variable	Cross-sectional analyses		Longitudinal analyses	
		*β* (95%CI)	*p-*value	*β* (95%CI)	*p-*value
1:4 PSM	Global Cognition	−0.47(− 0.69, − 0.25)	< 0.001	−0.22(− 0.44, − 0.01)	0.038
	Episodic Memory	−0.14(− 0.24, − 0.05)	0.004	−0.15(− 0.25, − 0.05)	0.003
	TICS	−0.22(− 0.38, − 0.05)	0.009	−0.04(− 0.21, 0.12)	0.590
80 < MCV < 100	Global Cognition	−0.48(− 0.71, − 0.24)	< 0.001	−0.31(− 0.54, − 0.08)	0.009
	Episodic Memory	−0.13(− 0.23, − 0.03)	0.014	−0.14(− 0.25, − 0.03)	0.016
	TICS	−0.20(− 0.37, − 0.03)	0.023	−0.10(− 0.27, 0.08)	0.266
BMI ≥ 18.5	Global Cognition	−0.42(− 0.63, − 0.21)	< 0.001	−0.22(− 0.42, − 0.02)	0.030
	Episodic Memory	−0.13(− 0.22, − 0.04)	0.005	−0.15(− 0.25, − 0.06)	0.002
	TICS	−0.17(− 0.32, − 0.02)	0.031	−0.03(− 0.19, 0.12)	0.653

*BMI* Body mass index, *MCV* Mean corpuscular volume, *eGFR* Estimated glomerular function rate, *CI* Confidence interval, *PSM* Propensity Scoring Matching, *TICS* Telephone Interview of Cognitive Status

*Notes:*^a^ Anemia was defined as hemoglobin concentrations lower than 13 g/dl for men and lower than 12 g/dl for women; All sensitivity analyses were conducted with the Cognitive Test Scores as the outcome and the baseline dichotomous anemia as the independent variable; All the analyses were adjusted for age, education level, marital status, BMI, cigarette smoking status, alcohol consumption, C reactive protein, total cholesterol, high density lipoprotein cholesterol, chronic pain, MCV, eGFR and associated comorbidities (cancer, kidney, stroke, heart, lung, hypertension, diabetes);

## Discussion

In this large representative sample of middle-aged and older Chinese, we found an independently association between anemia and baseline cognitive decline as well cognitive decline measured 2 years later after adjusting for a range of socio-demographic and health-related covariates. Besides, the global cognitive function was also found to have a linear relationship with hemoglobin concentration and the results showed the lower the hemoglobin concentrations, the worse the performance of cognitive function. Furthermore, the poor performance of episodic memory was found to be associated with anemia both cross-sectionally and longitudinally without gender difference and independent of covariates in this study.

The findings of our research are consistent with previous literatures which demonstrated the association between anemia and cognitive decline in the general population. For example, a research from the Atherosclerosis Risk in Communities (ARIC) study with a sample of 13,133 general participants (mean age 57 years and 56% women) found anemia was associated with partial cognitive domains- digit symbol substitution test (DSST) and global Z-score. However, this significant relationship was cross-sectional, not prospective. Moreover, they also were unable to find the linear association between hemoglobin level and worse cognition in either cross-sectional or longitudinal analyses [17]. Another prospective cohort study included 1744 community-dwelling participants (mean age 78 years and 65% women) found anemia was strongly associated with poor cognitive function, which has been measured by the Short Portable Mental Status Questionnaire (SPMSQ) [41] . In addition, one study with 180 elderly persons (mean age 71.5 years and 62.2% women) found that anemic persons had lower Mini-Mental State Examination (MMSE) scores than those non-anemic ones, and concluded that anemia may impair the cognitive functions in the elderly [42]. The significant association between lower hemoglobin concentrations and cognitive decline found in this study was also in line with previous findings. One study from Rush Memory and Aging Study reported that low hemoglobin concentrations were associated with worse performance on semantic memory and perceptual speed [43]. Another study from the same sample reported that lower hemoglobin levels were not only associated with a more rapid global cognitive decline, but also associated with an increased hazard for developing AD [44]. Previous studies together with the present study have proved that anemia or low hemoglobin concentration were associated with poor cognitive performances. Sensitivity analyses after excluding participants with low or high MCV as well as those with low BMI did not change the significant relationship between anemia and cognitive decline. This might suggest the relationship between anemia and cognitive decline could not influenced by iron deficiency, vitamin deficiency, or nutritional deficiency. Furthermore, the method of propensity score matching can perfectly balance the distribution of covariates between different target groups [45]. Thus, the significant association between anemia and cognition decline after propensity score matching indicated the influence of anemia on cognitive decline was independent of socio-demographic or health-related factors.

The relationship between anemia and cognitive decline seems to be mediated by inadequate cerebral oxygen delivery and inadequate cerebral oxygenation, which may reflect the impaired cerebral perfusion and cerebral function [46, 47]. Low concentrations of hemoglobin or anaemia can contribute chronic brain hypoxia and reduced aerobic capacity, thus increasing the risk of dementia or cognitive decline [48]. From the clinical point of view, brain ischemia is a known risk factor of cognitive dysfunction and dementia [49]. Animal models showed that erythropoietin receptors were localized in the brain and play a role in neuroprotection [50, 51]. The decreased erythropoietin levels, which could be induced by hypoxia, may increase the risk of neuronal degeneration in certain cognitive pathways. Besides, the appropriate higher hemoglobin concentration level means adequate oxygen delivery. So, as we found in the analysis, the higher hemoglobin concentration levels were slightly associated with better cognitive performance. Moreover, observational studies have reported that the use of erythropoiesis stimulating agents can improve the cognitive functions among patients with anemia [52–54].

Episodic memory is an important component in the measurement of the cognitive function. It’s a kind of declarative memory which belongs to long-term memory and presents a cognitive system that enables an individual to record, store, and retrieves information about personal experiences and the temporal and spatial contexts of those experiences [55]. The decline of episodic memory is the hallmark feature of early stages of AD [56]. In the processes of episodic memory decline, a vast cerebral network is involved, such as decreased in white and gray matter volumes, neuronal numbers and size, reduced efficiency of synaptic contacts, and decreased in the concentrations of neurotransmitters [57]. Although these changes may increase the risk of developing AD, they can also remain stable for many years or even revert to a cognitively normal state over time. This modifiable characteristic makes the slight cognitive change a promising target in the prevention of dementia.

Consistent with other prospective analyses which have found significant longitudinal associations between anemia and cognitive decline, our research also showed that anemia was associated with the longitudinal cognitive decline 2 years later [15, 17, 44]. In addition, the results showed women and men shared the same risk of cognitive decline, especially the episodic memory decline when they were anemic. The absence of gender difference was also consistent with prior studies [19, 58]. However, given the TICS score was measured only by some components of TICs battery, the observed association between anemia and lower TICS score was less significant among the men and the women. The significant association between anemia and cognitive decline among middle-aged and the elderly indicated that regardless of age and gender, there needs to be early intervention in regards to anemia to prevent pathological cognitive decline.

Some strengths of the present study deserve to be mentioned. First, the sample size of CHARLS was relatively large and the participants were from 150 county units, containing extensive areas and diverse population, having a very good representativeness of the Chinese population. To the best of our knowledge, this is the first try to test the association between anemia and specific cognitive domains in Chinese population. The results provide supplementary evidences to further investigate the underlying relationship between anemia and cognitive decline. Besides, the sample was relatively younger (mean age 59.13 years) than in other studies and the positive results provided more useful information indicating that not only the elderly, but also the middle aged adults were faced with the threat of anemia when aging. Secondly, we have comprehensively measured a broad range of potential confounders known to affect the hemoglobin concentrations and the cognitive function (including inflammatory markers, renal function and the comorbidities assessed in the baseline of our study) and were adequately adjusted in our step-wise analyses. Thirdly, apart from the dichotomous anemia variable, the continuous hemoglobin levels were also used in this study to further explore the relationship with anemia and the results showed lower hemoglobin was associated with worse cognitive function. Utilizing both dichotomous and continuous measures was a better way to characterize the relationship between anemia and cognitive function. Lastly, we conducted three kinds of sensitivity analyses to take different potential conditions into consideration to make full use of information of the data. The results were stable and convincible.

Meanwhile, certain limitations of our study should also be taken into consideration. The iron, vitamin B_12_, plasma homocysteine, folic acid and thyroid hormone levels are important indicators to estimate anemia, but the lack of assessment of these variables in CHARLS made us unable to specify the subtype of anemia or adjust them in this study. In addition, the hemoglobin concentrations were just detected once at baseline, so we cannot consider the change of hemoglobin during longitudinal analysis. Besides, just one-time assessment of hemoglobin concentration may not be an accurate estimate of an individuals’ typical concentration levels, it would be better if there were more times assessments of hemoglobin concentration. Although the episodic memory and intact mental status can be used to represent the majority domains of cognitive functions, according to the definition of cognitive function from the Oxford dictionary - “the mental action or process of acquiring knowledge and understanding through thought, experience, and the sense” [59], the cognitive domain of mental status and intactness measured in our study were relatively limited (just measured by some components of TICs battery), this may be the main reason that there were less significant findings observed for this cognitive domain.

## Conclusions

In conclusion, our study has found that anemia was associated with poor cognitive performance both cross-sectionally and longitudinally among Chinese middle-aged and elderly without gender specific difference. The lower hemoglobin concentrations were associated with linear cognitive decline. Anemia is relatively common and concealed disease among adults and has been neglected for a long time. In this case, effective intervention strategies for anemia should be implemented early to prevent its potential risk for cognitive decline. In future studies, more representative and large community-based cohorts with longer follow-up are needed to explore more modifiable risk factors to age-related cognitive decline, thus targeted multi-domain prevention approaches can benefit more middle-aged and elderly people.

## Data Availability

The datasets supporting the conclusions of this article are publicly available in the https://opendata.pku.edu.cn/dataverse/CHARLS.

## Abbreviations

CES-D 10: 10-item Center for Epidemiologic Studies Depression Scale score.
eGFR: Estimated glomerular function rate
Hs-CRP: High-sensitivity of C-reactive protein;
